# ﻿New species and records of *Hybos* Meigen (Diptera, Empidoidea) from Huaping National Nature Reserve, China

**DOI:** 10.3897/zookeys.1200.120258

**Published:** 2024-05-02

**Authors:** Meilin Li, Jingyu Wang, Ding Yang

**Affiliations:** 1 Department of Entomology, College of Plant Protection, China Agricultural University, Beijing 100193, China China Agricultural University Beijing China; 2 Comprehensive Technology Service Center of Rizhao Customs, Rizhao 276826, China Comprehensive Technology Service Center of Rizhao Customs Rizhao China

**Keywords:** Checklist, hybotid flies, key, new species, newly recorded species, South China region, taxonomy

## Abstract

In this study, 21 species of *Hybos* Meigen, 1803 are reviewed in Huaping National Nature Reserve, China. Among these, three species, i.e., *Hybosdenticulatus***sp. nov.**, *Hybosforcipata***sp. nov.** and *H.paraterminalis***sp. nov.**, are described as new to science. In addition, nine known species of this genus are reported for the first time in Guangxi. All the known species were enumerated, and an identification key to the species of *Hybos* from Huaping National Nature Reserve based on morphological characteristics is provided.

## ﻿Introduction

*Hybos* Meigen, 1803 is a species-rich genus of Empidoidea occurring worldwide. To date, 242 species of *Hybos* have been recorded worldwide, of which 28 species are distributed in the Palaearctic Realm and 191 species are distributed in the Oriental Realm (Yang and Yang 2004; Yang et al. 2007; Plant 2013; Shamshev et al. 2013; Shamshev et al. 2015; Li et al. 2017; Cao et al. 2018; Kanavalová et al. 2021; Li et al. 2022; Li and Yang 2023).

Huaping National Nature Reserve, the oldest national-level nature reserve established in Guangxi, has rich and diverse animal and plant resources and is an important gene bank of biological species in China. The climate here is humid, with relative humidity ranging from 85% to 90% during the rainy season from April to August, and a forest coverage rate of 98.2%, providing a relatively suitable environment for the survival of *Hybos*. During the collection investigation of Huaping National Nature Reserve in June 1982, five new *Hybos* species were discovered and reported (*H.ensatus* Yang & Yang, 1986, *H.flaviscutellum* Yang & Yang, 1986, *H.longshengensis* Yang & Yang, 1986, *H.orientalis* Yang & Yang, 1986, *H.truncatus* Yang & Yang, 1986), showcasing its rich biodiversity (Yang and Yang, 2004).

We surveyed the insect diversity in the national nature reserve twice in 2023 to update the faunal information of the South China region. In this study, three new species, *H.denticulatus* sp. nov., *H.forcipata* sp. nov. and *H.paraterminalis* sp. nov., are reported and described. While, nine species, *H.bawanglingensis* Yang, 2008, *H.fujianensis* Li & Yang, 2023, *H.guizhouensis* Yang & Yang, 1988, *H.jianyangensis* Yang & Yang, 2004, *H.leucopogus* Li & Yang, 2023, *H.obtusatus* Yang & Grootaert, 2005, *H.particularis* Yang, Yang & Hu, 2002, *H.pingbianensis* Yang & Yang, 2004 and *H.xiaohuangshanensis* Yang, Gaimari & Grootaert, 2005 are newly recorded in Guangxi.

Diagnosis and figures are provided for all 21 species, including related known ones (*H.anae* Yang & Yang, 2004, *H.chinensis* Yang & Yang, 2004, *H.ensatus* Yang & Yang, 1986, *H.flaviscutellum* Yang & Yang, 1986, *H.longshengensis* Yang & Yang, 1986, *H.orientalis* Yang & Yang, 1986 and *H.truncatus* Yang & Yang, 1986). Further, the male genitalia of *H.bawanglingensis* and *H.nasutus* Yang & Yang, 1986 are re-illustrated, and 13 known species are photographed (*H.bawanglingensis*, *H.ensatus*, *H.fujianensis*, *H.guizhouensis*, *H.jianyangensis*, *H.longshengensis*, *H.nasutus*, *H.obtusatus*, *H.orientalis*, *H.particularis*, *H.pingbianensis*, *H.truncatus* and *H.xiaohuangshanensis*). A checklist and key of *Hybos* from Huaping National Nature Reserve are also provided.

## ﻿Material and methods

Material for this study were collected by sweeping in Huaping National Nature Reserve, Guangxi in May and August 2023. All the studied specimens are preserved in 80% ethanol and deposited in the Entomological Museum of China Agricultural University (CAU), Beijing.

Specimens were examined using a ZEISS Stemi 2000c. Images were made by connecting the microscope with a Canon EOS 5D Mark IV camera. Image plates were post-processed with Adobe Photoshop CS6 Extended. Representative specimens were dissected. Male external genitalia were drawn after macerating the apical portion of the abdomen with cold 20% hydroxide (NAOH) for 4–8 h. Species of *Hybos* from China have been thoroughly reviewed and keyed (Yang and Yang 2004), providing us with a useful tool to identify the species in this study.

Abbreviations and morphological terms used in the text: **acr**–acrostichal bristle(s), **ad**–anterodorsal bristle(s), **av**–anteroventral bristle(s), **dc**–dorsocentral bristle(s), **ppn**–postpronotal humeral bristle(s), **npl**–notopleural bristle(s), **oc**–ocellar bristle(s), **pd**–posterodorsal bristle(s), **prsc**–prescutellar bristle(s), **psa**–postalar bristle(s), **pv**–posteroventral bristle(s), **sc**–scutellar bristle(s).

## ﻿Taxonomy

### ﻿Family Hybotidae

Taxon classificationAnimaliaDipteraHybotidae

Meigen, 1820

0D8BCA01-AA89-5BCF-928E-4DEBDAC10C30

Hybotinae Meigen, 1820: x. Type genus Hybos Meigen, 1803.
Hybotidae Macquart, 1827: 136.

### ﻿Genus Hybos

Taxon classificationAnimaliaDipteraHybotidae

Meigen, 1803.

42E5402E-91E6-5513-896E-CDD40BCB390C

Hybos Meigen, 1803: 269. Type species: Hybosfunebris Meigen, 1804.
Neoza Meigen, 1800: 27. Type species: Muscagrossipes Linnaeus, 1767.
Pseudosyneches Frey, 1953: 66. Type species: Hybos (Pseudosyneches) palawanus Frey, 1953.

#### Diagnosis.

*Hybos* is distinguished from all other Empidoidea genera by the following combination of characters: (1) vein Rs short arising distal to the middle of cell bm; (2) cell cup usually distinctly longer than bm; (3) eyes narrowly but distinctly separated on face, not virtually contiguous; (4) proboscis narrow, long spine-like, as long as head or longer, lacking pseudotracheae; (5) hind femur usually strongly thickened with strong ventral bristles; and (6) hind tibia linear (apart from basal geniculation) or slightly thickened apically.

### ﻿Key to species of *Hybos* from Huaping National Nature Reserve

This key is used for identifying *Hybos* in Huaping National Nature Reserve. Users are urged to confirm all decisions by referring to detailed descriptions. There are likely to be other undiscovered new species in Huaping National Nature Reserve. Therefore, it needs to be used with caution.

**Table:** 

1	All legs uniformly dark brown to black excluding hind knee	**2**
–	Legs at least partly yellow to yellow-brown excluding hind knee	**9**
2	All legs uniformly black-brown to black including hind knee	**3**
–	Legs dark brown to blackish, but only hind knee dark yellow	***H.fujianensis* Li & Yang, 2023**
3	Hind tibia apically without one pd and one av	**4**
–	Hind tibia apically with one pd and one av	***H.paraterminalis* sp. nov.**
4	Hind tibia without distinct bristles	**5**
–	Hind tibia with one dorsal bristle near apex	**7**
5	Mid tibia with one or two dorsal bristles	**6**
–	Mid tibia with four dorsal bristles	***H.jianyangensis* Yang & Yang, 2004**
6	R_4+5_ and M_1_ nearly parallel apically; mid tibia with two long dorsal bristles on basal ½	***H.anae* Yang & Yang, 2004**
–	R_4+5_ and M_1_ weakly convergent apically; mid tibia with one very long dorsal bristle at middle	***H.leucopogus* Li & Yang, 2023**
7	Mid femur with ad and pv	8
–	Mid femur only with pv	***H.obtusatus* Yang & Grootaert, 2005**
8	Hypandrium with row of long bristles near apical margin	***H.denticulatus* sp. nov.**
–	Hypandrium without long bristles near apical margin	***H.forcipata* sp. nov.**
9	Fore and mid femora brownish to black	**10**
–	Fore and mid femora uniformly or mostly yellow	**15**
10	Mid tibia black-brown to black	**11**
–	Mid tibia yellow to brownish	**12**
11	Hind knee black-brown and fore tibia only with one dorsal bristle at middle	***H.ensatus* Yang & Yang, 1986**
–	Hind knee yellow and fore tibia with four to five dorsal bristles	***H.xiaohuangshanensis* Yang, Gaimari & Grootaert, 2005**
12	Legs uniformly brownish	***H.truncatus* Yang & Yang, 1986**
–	Legs partly brownish	**13**
13	Fore tarsomeres 1–2 yellow	***H.guizhouensis* Yang & Yang, 1988**
–	Fore tarsomeres 1–2 black-brown to black	**14**
14	Left surstylus with two processes	***H.longshengensis* Yang & Yang, 1986**
–	Left surstylus with three processes	***H.particularis* Yang, Yang & Hu, 2002**
15	Fore and mid femora uniformly yellow including dorsally	**16**
–	Fore and mid femora mostly yellow except dark yellow-brown dorsally	***H.serratus* Yang & Yang, 1992**
16	Hind femur black-brown to black	**17**
–	Hind femur mostly yellow	**18**
17	Fore coxa black-brown	***H.chinensis* Yang & Yang, 2004**
–	Fore coxa yellow	***H.pingbianensis* Yang & Yang, 2004**
18	Hind tibia with one dorsal bristle at middle	**19**
–	Hind tibia without dorsal bristles at middle	***H.flaviscutellum* Yang & Yang, 1986**
19	Arista with short pubescence	**20**
–	Arista bare	***H.nasutus* Yang & Yang, 1986**
20	Right surstylus furcated, with three processes	***H.bawanglingensis* Yang, 2008**
–	Right surstylus triangular, without processes	***H.orientalis* Yang & Yang, 1986**

### ﻿Checklist of *Hybos* in Huaping National Nature Reserve of China

New records in Guangxi in bold

*Hybosanae* Yang & Yang, 2004 (Fujian, Guangxi)

*Hybosbawanglingensis* Yang, 2008 (**Guangxi**, Hainan)

*Hyboschinensis* Yang & Yang, 2004 (Fujian, Guangxi, Guizhou, Zhejiang)

*Hybosdenticulatus* sp. nov. (Guangxi)

*Hybosensatus* Yang & Yang, 1986 (Guangxi, Guizhou, Henan, Sichuan)

Hybosflaviscutellum Yang & Yang, 1986 (Guangxi, Zhejiang)

*Hybosforcipata* sp. nov. (Guangxi)

Hybosfujianensis Li & Yang, 2023 (Fujian, **Guangxi**)

*Hybosguizhouensis* Yang & Yang, 1988 (**Guangxi**, Guizhou)

*Hybosjianyangensis* Yang & Yang, 2004 (Fujian, **Guangxi**, Guizhou, Zhejiang)

*Hybosleucopogus* Li & Yang, 2023 (Fujian, **Guangxi**)

*Hyboslongshengensis* Yang & Yang, 1986 (Fujian, Guangxi)

*Hybosnasutus* Yang & Yang, 1986 (Guangxi)

*Hybosobtusatus* Yang & Grootaert, 2005 (Guangdong, **Guangxi**, Guizhou)

*Hybosorientalis* Yang & Yang, 1986 (Fujian, Guangxi, Henan)

Hybosparaterminalis sp. nov. (Guangxi)

*Hybosparticularis* Yang, Yang & Hu, 2002 (**Guangxi**, Hainan)

*Hybospingbianensis* Yang & Yang, 2004 (**Guangxi**, Yunnan)

*Hybosserratus* Yang & Yang, 1992 (Fujian, Guangxi, Gzuihou, Henan, Sichuan, Yunnan, Zhejiang; Thailand)

Hybostruncatus Yang & Yang, 1986 (Guangxi)

*Hybosxiaohuangshanensis* Yang, Gaimari & Grootaert, 2005 (Fujian, Guangdong, **Guangxi**)

### ﻿ Hybos anae

Taxon classificationAnimaliaDipteraHybotidae

Yang & Yang, 2004

31AC1F96-B2CC-542F-8137-F8A9270BEB7C

Fig. 1

Hybos anae Yang & Yang, 2004: 124.

#### Type locality.

China: Guangxi, Longsheng.

#### Diagnosis.

Legs entirely black-brown. R_4+5_ and M_1_ nearly parallel apically. Hypandrium shallowly incised apically, with one long thick finger-like right process, bifurcated apically, and small subtriangular left process.

**Figure 1.**
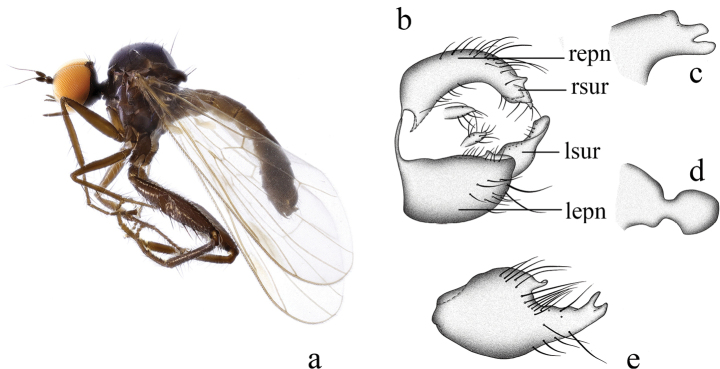
*Hybosanae***a** male habitus, lateral view **b** genitalia, dorsal view **c** right surstylus **d** left surstylus **e** hypandrium, ventral view (after Li and Yang 2023). Abbreviations: lepn = left epandrial lamella; lsur = left surstylus; repn = right epandrial lamella; rsur = right surstylus.

#### Distribution.

China (Fujian, Guangxi).

### ﻿ Hybos bawanglingensis

Taxon classificationAnimaliaDipteraHybotidae

Yang, 2008

1C0CB951-1038-5645-9FAD-11CD1BE1DACB

Fig. 2

Hybos bawanglingensis Yang, 2008: 618.

#### Type locality.

China: Hainan, Bawangling.

#### Material examined.

China • 2♂ 1♀, Guangxi, Guilin, Huaping, Tianpingshan; 770 m, 1 June 2023; Wei Zeng; CAU. China • 1♂ 3♀, Guangxi, Laibin, Dayaoshan, Shengtangshan; 1434 m, 14 August 2023; Wenqiang Cao; CAU.

**Figure 2.**
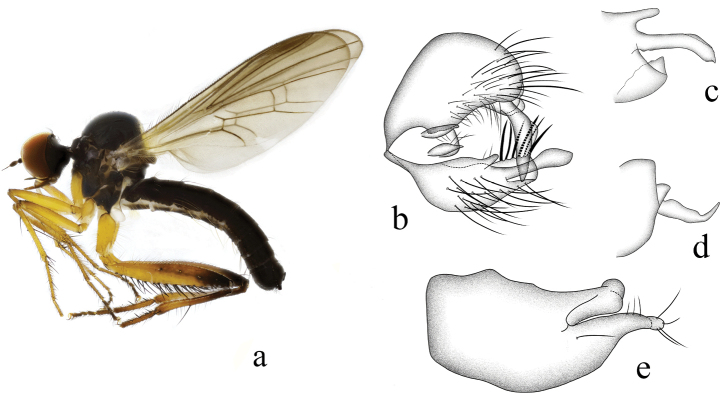
*Hybosbawanglingensis***a** male habitus, lateral view **b** genitalia, dorsal view **c** right surstylus **d** left surstylus **e** hypandrium, ventral view

#### Diagnosis.

Legs yellow except hind knee dark brown, tarsomeres 3–5 black. Hind tibia with one ad at middle. Hypandrium with a narrow cleft apically.

#### Distribution.

China (Guangxi, Hainan).

### ﻿ Hybos chinensis

Taxon classificationAnimaliaDipteraHybotidae

Yang & Yang, 2004

D2EB1B77-DB26-5662-97B2-A3516D405ED8

Fig. 3

Hybos chinensis Frey, 1953: 64; Yang and Yang 2004: 143.

#### Type locality.

China: Fujian.

#### Material examined.

China • 6♂, Guangxi, Guilin, Huaping, Anjiangping; 1340 m, 26 May 2023; Wei Zeng; CAU.

#### Diagnosis.

Legs black-brown, except fore and mid knees, femora, tarsomeres 1–2 and all tibiae yellow; fore and mid tarsomeres 3–5 yellow-brown, hind tarsus yellow-brown. Hypandrium with small process on left corner.

**Figure 3.**
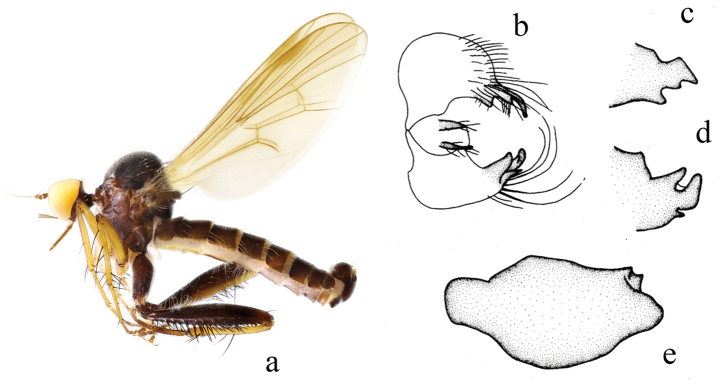
*Hyboschinensis***a** male habitus, lateral view **b** genitalia, dorsal view **c** right surstylus **d** left surstylus **e** hypandrium, ventral view (after Yang and Yang 2004)

#### Distribution.

China (Fujian, Guangxi, Guizhou, Zhejiang).

### ﻿ Hybos denticulatus sp. nov.

Taxon classificationAnimaliaDipteraHybotidae

CB50AEEA-D648-53BB-92D3-F0B485C30F0F

https://zoobank.org/416CDB05-FFAF-415A-BA07-45606E7C65F1

Fig. 4

#### Type material examined.

***Holotype***: China •♂; Guangxi, Guilin, Huaping, Anjiangping; (25°33'44.2"N, 109°56'42.4"E, 1340 m), 28 May 2023, Wei Zeng; CAU.

#### Diagnosis.

Legs entirely black. Hind tibia with one ad near apex. R_2+3_ curved, R_4+5_ and M_1_ slightly convergent apically. Hypandrium with row of long bristles near apical margin.

#### Description.

**Male.** Body length 4.7 mm. Wing length 4.3 mm.

***Head*** black with gray pollen. Eyes contiguous on frons, black-brown with slightly enlarged dorsal facets yellow-brown. Hairs and bristles on head black except posteroventral surface with partly dark brown hairs; ocellar tubercle distinct with two long oc and two short posterior hairs. Antenna black; scape without hairs, pedicel with circlet of blackish subapical hairs; first flagellomere blackish, not elongated, nearly as long as scape and pedicel combined, without dorsal hairs; arista blackish, short pubescent except apical ¼ or so thin and bare. Proboscis shorter than head, black. Palpus blackish, with one blackish apical hair.

***Thorax*** black with gray pollen. Hairs on thorax blackish, bristles black; hairs on mesonotum slightly long, ppn absent, two npl (anterior npl rather short), uniserial hair–like dc nearly as long as irregularly quadriserial acr, two prsc, one psa; scutellum with eight marginal hairs and two sc. Legs entirely black. Hairs on legs mostly dark brown to blackish, bristles black-brown to black, but those on coxae partly brownish. Fore femur 1.3× and hind femur 1.9× as wide as mid femur. Fore femur with row of pv distinctly longer than femur thickness. Mid femur with 3–4 ad on basal ⅓ and row of pv distinctly longer than femur thickness; apically with one weak ad. Hind femur with row of ad on apical 2/3, ~ three rows of spine-like ventral bristles on tubercles and some dorsal hairs on basal 1/5. Fore tibia with row of short or slightly long ad and some long thin pv hairs; apically with 4 bristles including one thick ad. Mid tibia with row of thin or slightly thick ad; apically with one long av. Hind tibia with one ad near apex. Fore tarsomere 1 with some long ad and pv hairs. Mid tarsomere 1 with one ad near middle; apically with one slightly long ad. Hind tarsomere 1 with short dense spine-like ventral bristles. Wing hyaline, stigma dark brown; veins brown to black-brown, R_2+3_ curved, R_4+5_ and M_1_ slightly convergent apically. Squama dark yellow with dark yellow hairs. Halter dark yellow with dark brown stem and pale-yellow knob.

***Abdomen*** short thick, black with pale gray pollen, hypopygium slightly thicker than pregenital segments. Hairs and bristles on abdomen yellow-brown to brown except those on hypopygium black.

**Figure 4.**
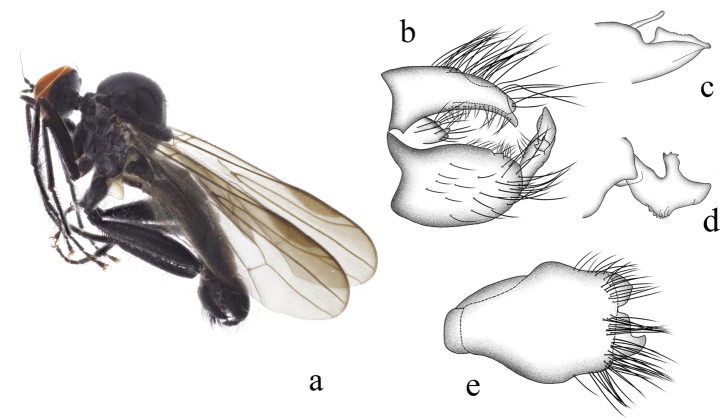
*Hybosdenticulatus* sp. nov. **a** male habitus, lateral view **b** genitalia, dorsal view **c** right surstylus **d** left surstylus **e** hypandrium, ventral view

***Male genitalia.*** Left epandrial lamella distinctly wider than right epandrial lamella (Fig. 4b); left surstylus with wide finger-like process, right lateral margin with one process, left lateral margin with some middle denticles (Fig. 4d). Right epandrial lamella with concave inner margin; right surstylus with long wide subtriangular process, lateral margin with one thin finger-like process apically (Fig. 4c). Hypandrium ~ 1.5× longer than wide, narrow basally and wide apically, apical margin with two wide processes, with row of long bristles near apical margin (Fig. 4e).

**Female.** Unknown.

#### Etymology.

This specific name refers to the left surstylus with some middle denticles on the lateral margin.

#### Distribution.

China (Guangxi).

#### Remarks.

The new species is similar to *H.brevis* Yang & Yang from Zhejiang, 1995, but may be separated by the arista and the left surstylus. In the new species, the arista is short pubescent, and the left surstylus has some middle denticles on the lateral margin. In *H.brevis*, the arista is bare, and the left surstylus lacks denticles (Yang and Yang 2004).

### ﻿ Hybos ensatus

Taxon classificationAnimaliaDipteraHybotidae

Yang & Yang, 1986

85C61305-4B5A-570A-B283-73863EE08CF7

Fig. 5

Hybos ensatus Yang & Yang, 1986: 83; Yang and Yang 2004: 155.

#### Type locality:

China: Guangxi, Longsheng.

#### Diagnosis.

Legs black-brown, except mid tarsi yellow-brown. Mid tibia with 2 long bristles on basal half. Male genitalia: left epandrial lobe with process at inner margin near middle; right surstylus sword-shaped.

**Figure 5.**
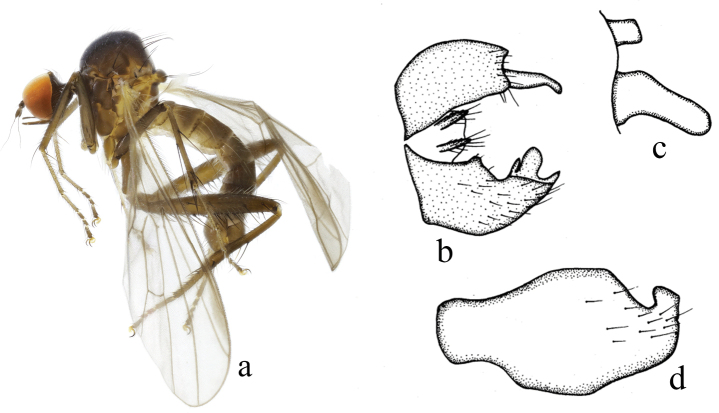
*Hybosensatus***a** male habitus, lateral view **b** genitalia, dorsal view **c** right surstylus **d** hypandrium, ventral view. (b–d: after Yang and Yang 2004)

#### Distribution.

China (Guangxi, Guizhou, Henan, Sichuan).

### ﻿ Hybos flaviscutellum

Taxon classificationAnimaliaDipteraHybotidae

Yang & Yang, 1986

1ABA61C2-2FB5-568E-809F-854EB4F13515

Fig. 6

Hybos flaviscutellum Yang & Yang, 1986: 81; Yang and Yang 2004: 158.

#### Type locality.

China: Guangxi, Longsheng.

#### Diagnosis.

Scutellum yellow. Legs yellow to yellow-brown, except tarsomeres 3–5 dark yellow. Male genitalia: left epandrial lobe rather wide; left surstylus knife-shaped.

**Figure 6.**
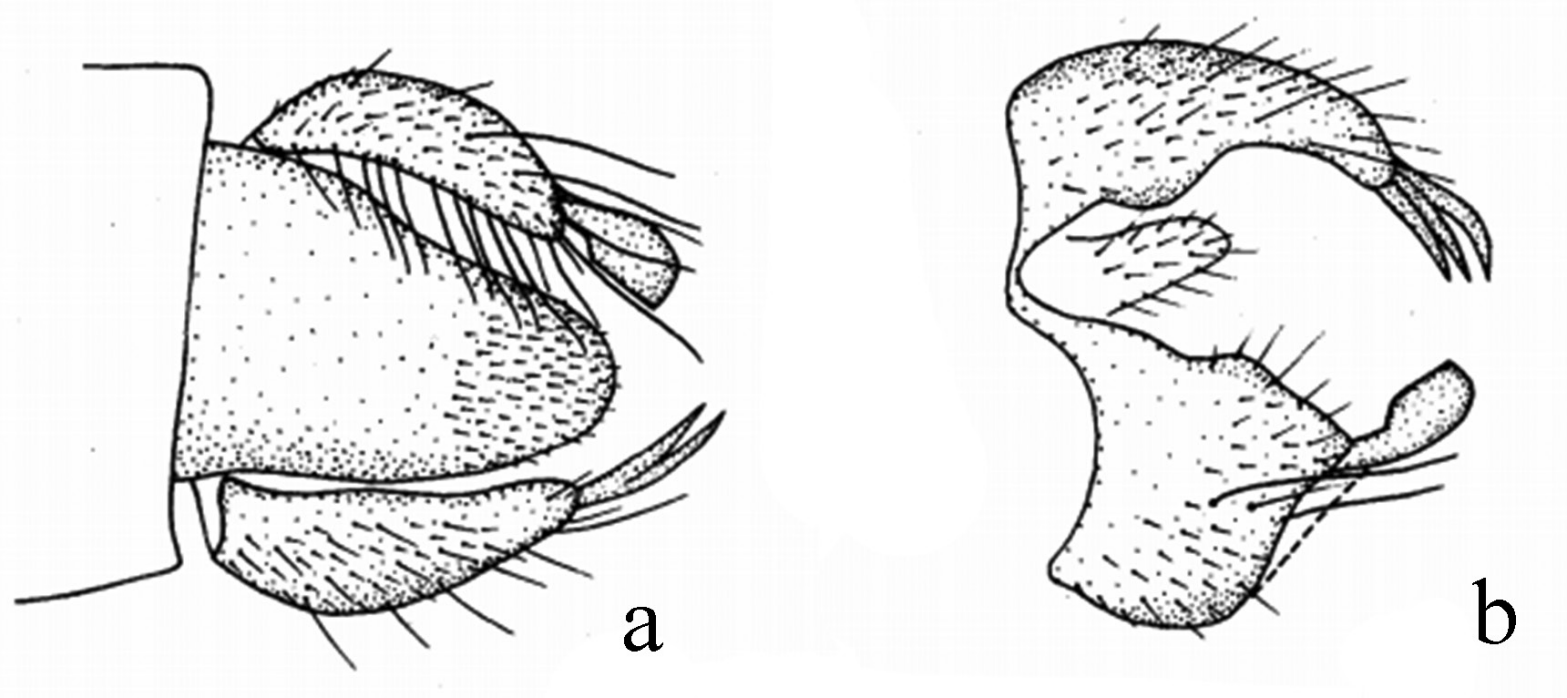
*Hybosflaviscutellum***a** hypandrium, ventral view **b** genitalia, dorsal view (after Yang and Yang 2004)

#### Distribution.

China (Guangxi, Zhejiang).

### ﻿ Hybos forcipata sp. nov.

Taxon classificationAnimaliaDipteraHybotidae

241670C2-4457-58B1-9E20-1BD8971EF1B1

https://zoobank.org/4CC29607-1E47-49BA-8153-8C19966E2407

Fig. 7

#### Type material examined.

***Holotype***: China •♂; Guangxi, Guilin, Huaping, Anjiangping; (25°33'42.0"N, 109°56'37.2"E, 1413 m), 7 August 2023, Wenqiang Cao; CAU.

#### Diagnosis.

Legs entirely black. Mid tarsomere 1 with one ad near apex and some short or long hairs. R_4+5_ and M_1_ slightly convergent apically. Left surstylus claw-shaped in lateral view.

#### Description.

**Male.** Body length 3.3 mm. Wing length 2.8 mm.

***Head*** black with gray pollen. Eyes contiguous on frons, black-brown with slightly enlarged dorsal facets yellow-brown. Hairs and bristles on head black except posteroventral surface with partly dark brown hairs; ocellar tubercle distinct with two very short hairs. Antenna blackish; scape without hairs, pedicel with circlet of black-brown subapical hairs; first flagellomere black-brown, slightly elongated, longer than scape and pedicel combined, without dorsal hairs; arista black-brown, short pubescent. Proboscis distinctly shorter than head, black-brown. Palpus blackish, with one black-brown apical hair.

***Thorax*** black with gray pollen. Hairs on thorax blackish, bristles black; hairs on mesonotum short, ppn absent, two npl (anterior npl rather short), uniserial hair–like dc nearly as long as irregularly quadriserial acr, two prsc, one psa; scutellum with eight marginal hairs and two sc. Legs entirely black. Hairs on legs mostly black-brown to black, bristles blackish to black, but those on coxae partly brown. Fore femur 1.5× and hind femur 2.4× as wide as mid femur. Fore femur with row of pv distinctly longer than femur thickness. Mid femur with row of ad on apical ⅓ and row of long thin pv distinctly longer than femur thickness. Hind femur with two ad on apical ½ and ~ three rows of long spine-like ventral bristles on tubercles. Fore tibia with some short or long ad and pv hairs. Mid tibia with two ad on basal ½ and some long hairs; apically with one very long av. Hind tibia with one ad near apex. Fore tarsomere 1 with some short or long ad and pv hairs. Mid tarsomere 1 with one ad near apex and some short or long hairs. Hind tarsomere 1 with row of short dense spine-like ventral bristles. Wing hyaline, stigma brownish; veins brownish to dark brown, R_4+5_ and M_1_ slightly convergent apically. Squama dark yellow with dark yellow hairs. Halter dark yellow with brown stem and pale-yellow knob.

***Abdomen*** black with pale gray pollen. Hairs and bristles on abdomen brown except those on hypopygium blackish. Hypopygium distinctly thicker than pregenital segments.

**Figure 7.**
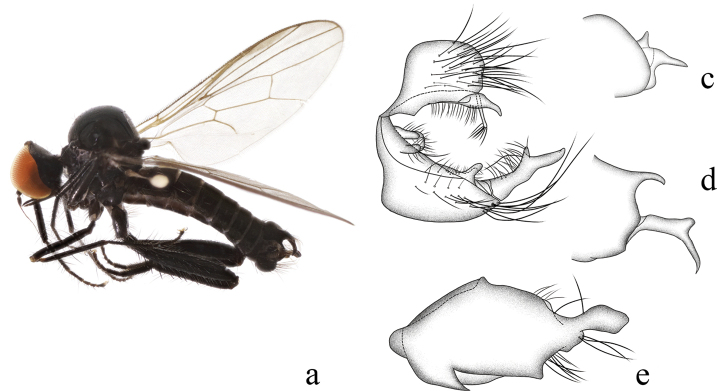
*Hybosforcipata* sp. nov. **a** male habitus, lateral view **b** genitalia, dorsal view **c** right surstylus **d** left surstylus **e** hypandrium, ventral view

***Male genitalia.*** Left epandrial lamella slightly narrower than right epandrial lamella, with inner margin obliquely subtruncate (Fig. 7b); left surstylus claw-shaped in lateral view; with one curved apical lateral process and one long process, furcated apically (Fig. 7d). Right epandrial lamella with weakly convex inner margin near middle; right surstylus furcated into one small triangular process and one finger-like process (Fig. 7c). Hypandrium ~ 2.2× longer than wide, narrow apically, right lateral margin with one trapezoid process and one triangle-like process (Fig. 7e).

**Female.** Unknown.

#### Etymology.

This specific name refers to the claw-shaped left surstylus, in lateral view.

#### Distribution.

China (Guangxi).

#### Remarks.

The new species is similar to *H.curvatus* Yang & Grootaert, 2005 from Guangdong, but may be separated by the form of the fore tibia and hypandrium. In the new species, the fore tibia bears some ad and pv hairs, and the hypandrium has two processes at lateral margin. In *H.curvatus*, the fore tibia has one av and one pv apically, and the hypandrium lacks processes on the lateral margin (Yang and Grootaert 2005).

### ﻿ Hybos fujianensis

Taxon classificationAnimaliaDipteraHybotidae

Li & Yang, 2023

60A5250C-3922-511C-B6B0-D3C34454F608

Fig. 8

Hybos fujianensis Li & Yang, 2023: 313–351

#### Type locality.

China: Fujian, Wuyishan.

#### Material examined.

China • 1♂, Guangxi, Guilin, Huaping, Anjiangping; 1413 m, 7 August 2023; Wenqiang Cao; CAU.

#### Diagnosis.

First flagellomere with two blackish dorsal hairs; arista bare. Legs mostly dark brown to black-brown. Hind tibia apically with long thin pd.

**Figure 8.**
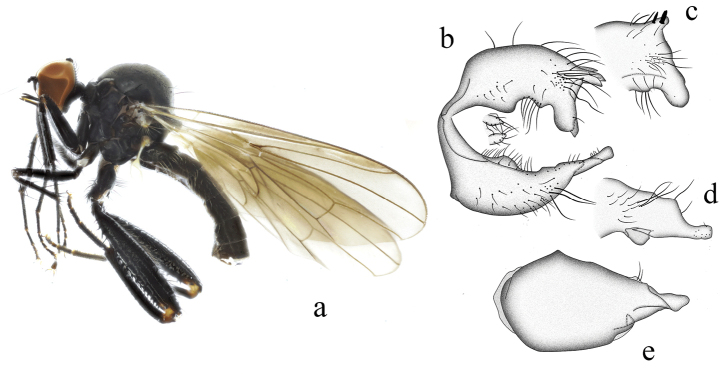
*Hybosfujianensis***a** male habitus, lateral view **b** genitalia, dorsal view **c** right surstylus **d** left surstylus **e** hypandrium, ventral view (**b–e**: after Li and Yang 2023)

#### Distribution.

China (Fujian, Guangxi).

### ﻿ Hybos guizhouensis

Taxon classificationAnimaliaDipteraHybotidae

Yang & Yang, 1988

9C1BB19A-966E-5F71-8D34-13347A7A8B13

Fig. 9

Hybos guizhouensis Yang & Yang, 1988: 136; Yang and Yang 2004: 168.

#### Type locality.

China: Guizhou, Fanjingshan.

#### Material examined.

China • 1♂, Guangxi, Guilin, Huaping, Hongtan; 849 m, 30 May 2023; Wei Zeng; CAU.

#### Diagnosis.

Legs brownish, except base of mid and hind tibia, fore and mid tarsomeres 1–2 yellow. Hypandrium with irregular process on apical margin.

**Figure 9.**
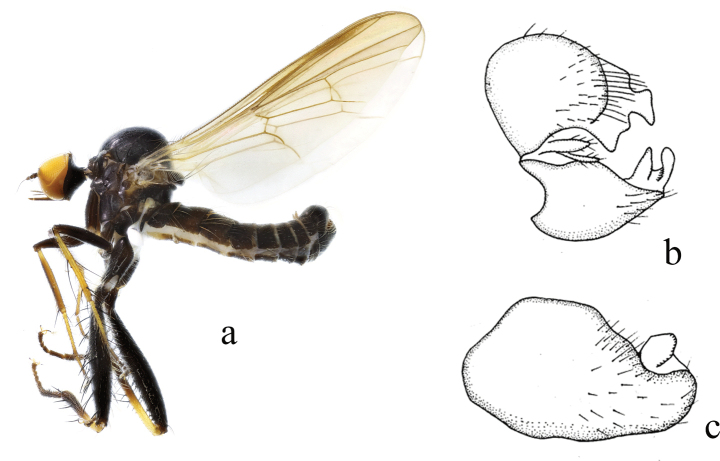
*Hybosguizhouensis***a** male habitus, lateral view **b** genitalia, dorsal view **c** hypandrium, ventral view (**b, c**: after Yang and Yang 2004)

#### Distribution.

China (Guangxi, Guizhou).

### ﻿ Hybos jianyangensis

Taxon classificationAnimaliaDipteraHybotidae

Yang & Yang, 2004

DB8D7430-10B0-5E6B-85ED-855D9CFF2166

Fig. 10

Hybos jianyangensis Yang & Yang, 2004: 178.

#### Type locality.

China: Fujian, Jianyang.

#### Material examined.

China • 2♂, Guangxi, Guilin, Huaping, Hongtan; 849 m, 30 May 2023; Wei Zeng; CAU.

#### Diagnosis.

Legs entirely black. Mid tibia with 4 dorsal bristles and 2 ventral bristles. Male genitalia: left surstylus rather wide with short finger-like inner lateral process.

**Figure 10.**
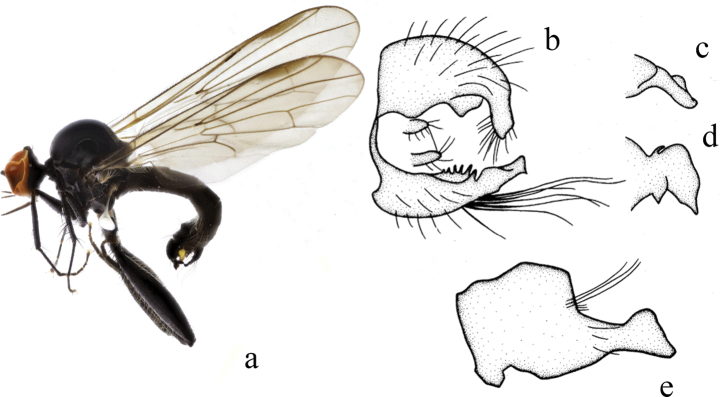
*Hybosjianyangensis***a** male habitus, lateral view **b** genitalia, dorsal view **c** right surstylus **d** left surstylus **e** hypandrium, ventral view (**b–e**: after Yang and Yang 2004)

#### Distribution.

China (Fujian, Guangxi, Guizhou, Zhejiang).

### ﻿ Hybos leucopogus

Taxon classificationAnimaliaDipteraHybotidae

Li & Yang, 2023

F8DBEC06-6CE9-5427-BDC8-974CCA6DBFEF

Fig. 11

Hybos fujianensis Li & Yang, 2023: 313–351

#### Type locality.

China: Fujian, Wuyishan.

#### Material examined.

China • 1♂ 1♀, Guangxi, Laibin, Dayaoshan, Yinshangongyuan; 1150 m, 15 August 2023; Wenqiang Cao; CAU.

#### Diagnosis.

Legs entirely black. Hind femur distinctly thickened. Hind tibia with one row of ad hairs and four pd hairs on basal ½. R_2+3_ weakly curved, R_4+5_ and M_1_ weakly convergent apically. Hypandrium narrow basally, bifurcated apically.

**Figure 11.**
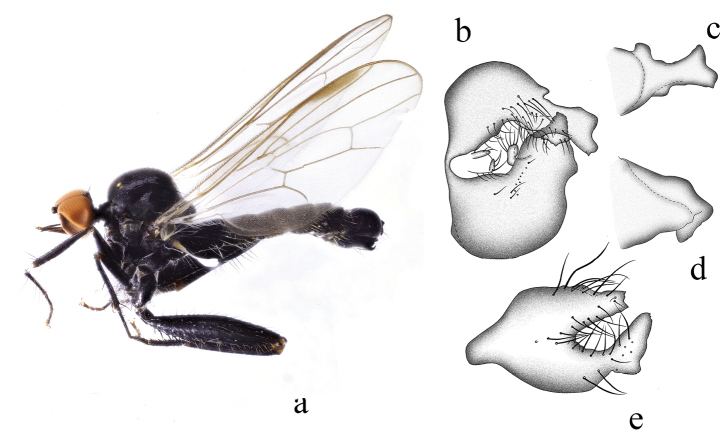
*Hybosleucopogus***a** male habitus, lateral view **b** genitalia, dorsal view **c** right surstylus **d** left surstylus **e** hypandrium, ventral view (after Li and Yang 2023)

#### Distribution.

China (Fujian, Guangxi).

### ﻿ Hybos longshengensis

Taxon classificationAnimaliaDipteraHybotidae

Yang & Yang, 1986

C89F3B4F-C50A-57C2-A1D3-C7C08D0F852B

Fig. 12

Hybos longshengensis Yang & Yang, 1986: 78; Yang and Yang 2004: 187.

#### Type locality.

China: Guangxi, Longsheng.

#### Diagnosis.

Arista bare. Legs black-brown, except mid tibia and tarsomeres 1–2 yellow, tips of hind femur, base and tips of tibia and all tarsi yellow. Hypandrium with right apical corner elongated outwards into one process.

**Figure 12.**
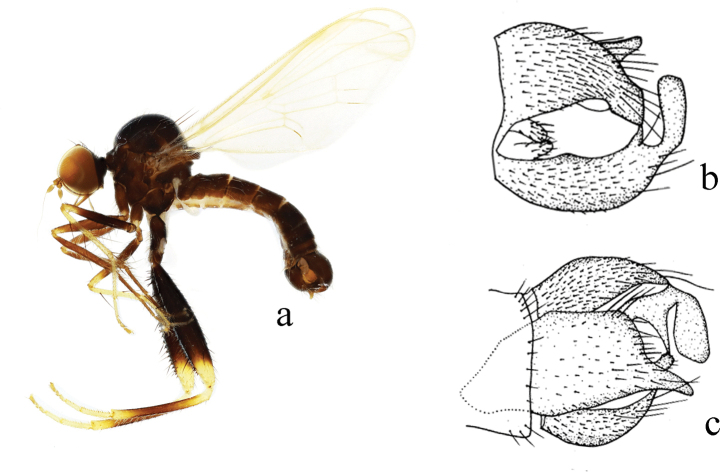
*Hyboslongshengensis***a** male habitus, lateral view **b** genitalia, dorsal view **c** hypandrium, ventral view (**b, c**: after Yang and Yang 2004)

#### Distribution.

China (Fujian, Guangxi).

### ﻿ Hybos nasutus

Taxon classificationAnimaliaDipteraHybotidae

Yang & Yang, 1986

45483903-0F4B-5C71-B77F-C2260A3024CF

Fig. 13

Hybos nasutus Yang & Yang, 1986: 79; Yang and Yang 2004: 197.

#### Type locality.

China: Guangxi, Jinxiu.

#### Material examined.

China • 4♂, Guangxi, Guilin, Huaping, Anjiangping; 1413 m, 7 August 2023; Wenqiang Cao; CAU. China • 3♂, Guangxi, Guilin, Huaping, Anjiangping; 1413 m, 7 August 2023, Wenqiang Cao; CAU.

**Figure 13.**
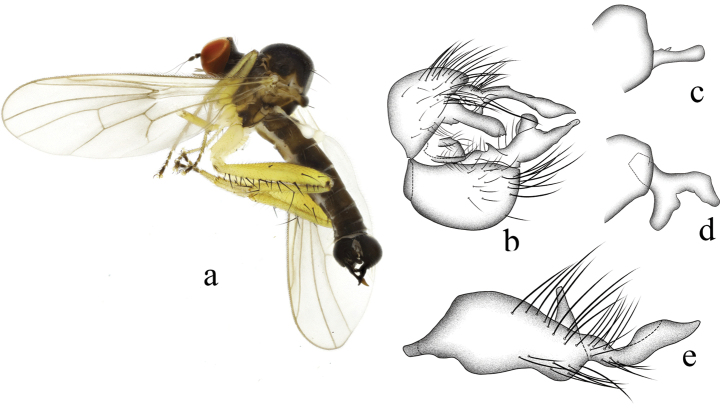
*Hyboslongshengensis***a** male habitus, lateral view **b** genitalia, dorsal view **c** right surstylus **d** left surstylus **e** hypandrium, ventral view

#### Diagnosis.

Arista bare. Legs yellow, except tarsomeres 3–5 dark yellow. Hind tibia with one dorsal bristle at middle; apically with one dorsal bristle and one ventral bristle.

#### Distribution.

China (Guangxi).

### ﻿ Hybos obtusatus

Taxon classificationAnimaliaDipteraHybotidae

Yang & Grootaert, 2005

61AA9DD7-A86E-5698-8A13-D8DB7368C62A

Fig. 14

Hybos obtusatus Yang & Grootaert, 2005: 410.

#### Type locality.

China: Guangdong.

#### Material examined.

China • 1♂, Guangxi, Guilin, Huaping, Anjiangping; 1340 m, 26 May 2023; Wei Zeng; CAU.

#### Diagnosis.

Palpus blackish with two long bristles at tip. Legs entirely black. R_4+5_ and M_1_ parallel apically.

**Figure 14.**
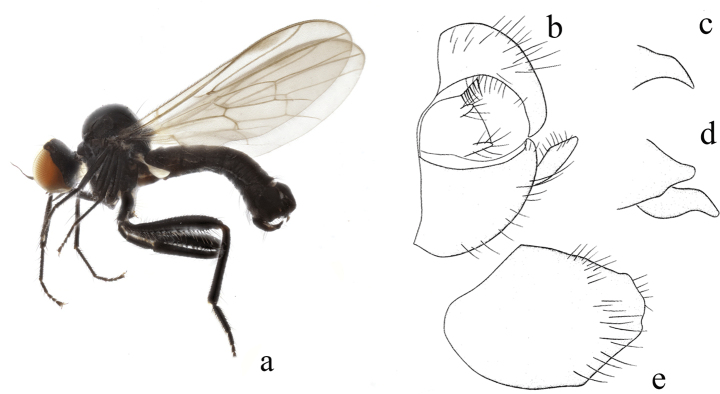
*Hybosobtusatus***a** male habitus, lateral view **b** genitalia, dorsal view **c** right surstylus **d** left surstylus **e** hypandrium, ventral view (**b–e**: after Yang and Grootaert 2005)

#### Distribution.

China (Guangdong, Guangxi, Guizhou).

### ﻿ Hybos orientalis

Taxon classificationAnimaliaDipteraHybotidae

Yang & Yang, 1986

CEF19C21-4F9E-5CB9-9D93-70AE7920CDB4

Fig. 15

Hybos orientalis Yang & Yang, 1986: 82; Yang and Yang 2004: 201.

#### Type locality.

China: Guangxi, Longsheng; Fujian, Jianyang.

#### Material examined.

China • 5♂5♀, Guangxi, Guilin, Huaping, Anjiangping; 1494 m, 7 August 2023; Wenqiang Cao; CAU. China • 5♂15♀, Guangxi, Guilin, Huaping, Anjiangping; 1514 m, 7 August 2023; Wenqiang Cao; CAU.

**Figure 15.**
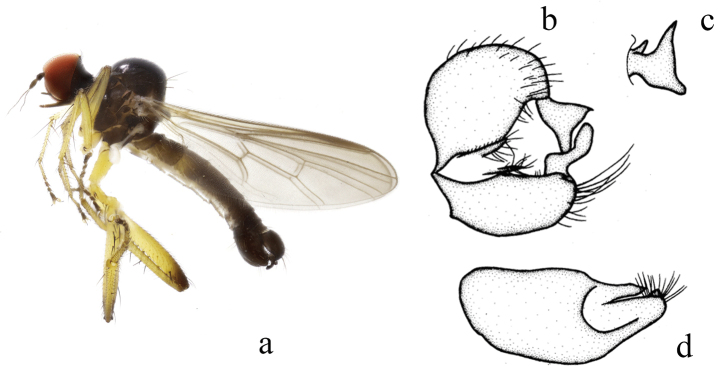
*Hybosorientalis***a** male habitus, lateral view **b** genitalia, dorsal view **c** right surstylus **d** hypandrium, ventral view (**b–d**: after Yang and Yang 2004)

#### Diagnosis.

Legs yellow, except tarsomeres 3–5 dark yellow and extreme tip of hind femur black. Hypandrium wide basally and small and obtuse apically.

#### Distribution.

China (Fujian, Guangxi, Henan).

### ﻿ Hybos paraterminalis sp. nov.

Taxon classificationAnimaliaDipteraHybotidae

FFA91AFC-D152-5B67-A161-E684A276821C

https://zoobank.org/2AB98B64-7EC8-4962-94E3-2F4DCFEAE076

Fig. 16

#### Type material examined.

***Holotype***: China •♂; Guangxi, Guilin, Huaping, Anjiangping; (25°33'39.8"N, 109°56'41.9"E, 1340 m), 26 May 2023, Wei Zeng; CAU.

#### Diagnosis.

Legs mostly black-brown to black except extreme base of hind femur and all tarsi brown to dark brown. Mid tarsomere 1 with two pv on basal ½. R_4+5_ and M_1_ divergent apically.

#### Description.

**Male.** Body length 4.2 mm. Wing length 4.3 mm.

***Head*** black with gray pollen. Eyes contiguous on frons, black-brown with distinctly enlarged dorsal facets yellow-brown. Hairs and bristles on head black except posteroventral surface with partly dark yellow hairs; ocellar tubercle indistinct. Antenna dark brown; scape without hairs, pedicel with circlet of brown subapical hairs; first flagellomere and arista absent. Proboscis slightly shorter than head, dark brown. Palpus dark brown, with one brown apical hair.

***Thorax*** black with gray pollen. Hairs on thorax blackish, bristles black; hairs on mesonotum short, ppn absent, two npl (anterior npl rather short), uniserial hair–like dc nearly as long as irregularly quadriserial acr, two long prsc, one psa; scutellum with 6 marginal hairs and two very long sc. Legs mostly black-brown to black except extreme base of hind femur and all tarsi brown to dark brown. Hairs on legs mostly brownish to dark brown, bristles black-brown to black, but those on coxae partly dark yellow, fore and mid femora with brownish bristles and hind femur with partly dark yellow hairs and bristles. Fore femur 1.2× and hind femur 1.6× as wide as mid femur. Fore femur with row of weak pv shorter than femur thickness. Mid femur with row of weak pv; apically with one ad. Hind femur with 4 ad on apical ½, ~ two rows of long spine-like ventral bristles on tubercles and row of long thin outer pv on apical ½. Fore tibia with one short ad near middle; apically with one ad. Mid tibia with one very long ad at apical ⅓, one very long av near middle; apically with 5 bristles including one rather long av. Hind tibia with two ad near middle; apically with one pd and one short av. Fore tarsomere 1 with one pv at extreme base. Mid tarsomere 1 with two pv on basal ½; apically with circle of bristles including one pv. Hind tarsomere 1 with row of short spine-like ventral bristles. Wing hyaline, stigma dark brown; veins brown to black-brown, R_4+5_ and M_1_ divergent apically. Squama dark yellow with dark yellow hairs. Halter dark yellow with brownish stem and pale-yellow knob.

***Abdomen*** black with pale gray pollen. Hairs and bristles on abdomen dark yellow to brownish except those on hypopygium blackish. Hypopygium distinctly thicker than pregenital segments.

**Figure 16.**
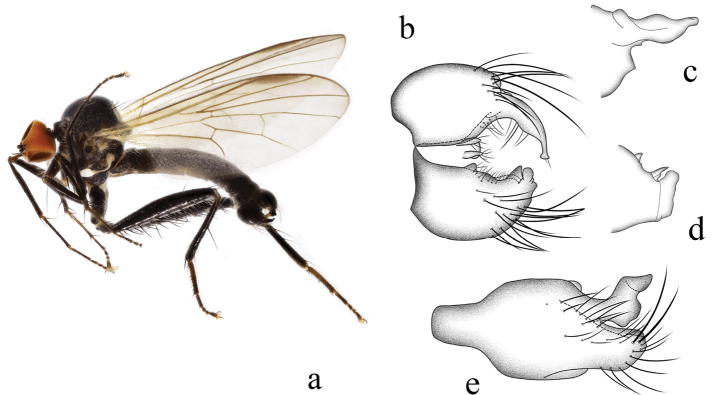
*Hybosparaterminalis* sp. nov. **a** male habitus, lateral view **b** genitalia, dorsal view **c** right surstylus **d** left surstylus **e** hypandrium, ventral view.

***Male genitalia.*** Left epandrial lamella as wide as right epandrial lamella, with inner margin slightly convex medially (Fig. 16b); left surstylus with apical margin very wide, truncate, apico–lateral portion with one small subtriangular process (Fig. 16d). Right epandrial lamella with concave inner margin near apex; right surstylus slightly wider at middle, long narrow apical portion (Fig. 16c). Hypandrium ~ 2.0× longer than wide, bilobate apically (left process wide and irregular in shape; right process wide finger-like, straight) (Fig. 16e).

**Female.** Unknown.

#### Etymology.

This specific name refers to the left surstylus with the very wide and truncate apical margin.

#### Distribution.

China (Guangxi).

#### Remarks.

The new species is similar to *H.guizhouensis* Yang & Yang, 1988 from Guizhou, but may be separated by having all tarsi brown to dark brown and the right surstylus slightly wider in the middle and a long narrow tip. In *H.guizhouensis*, the fore and mid tarsomeres 1–2 are yellow; and the right surstylus is narrow in the middle and slightly wider at the tip (Yang and Yang 2004).

### ﻿ Hybos particularis

Taxon classificationAnimaliaDipteraHybotidae

Yang, Yang & Hu, 2002

02A7BFEB-5BFD-5E47-B3B0-F7DA2E608AE2

Fig. 17

Hybos particularis Yang, Yang & Hu, 2002: 734; Yang and Yang 2004: 205.

#### Type locality.

China: Hainan, Jianfengling.

#### Material examined.

China • 1♂, Guangxi, Guilin, Huaping, Tianpingshan; 542 m, 4 August 2023; Wenqiang Cao; CAU.

#### Diagnosis.

Legs black, except tips of mid femur brownish, mid tibia and tarsomeres 1–2 yellow. Hypandrium long narrow, apically with deep incision.

**Figure 17.**
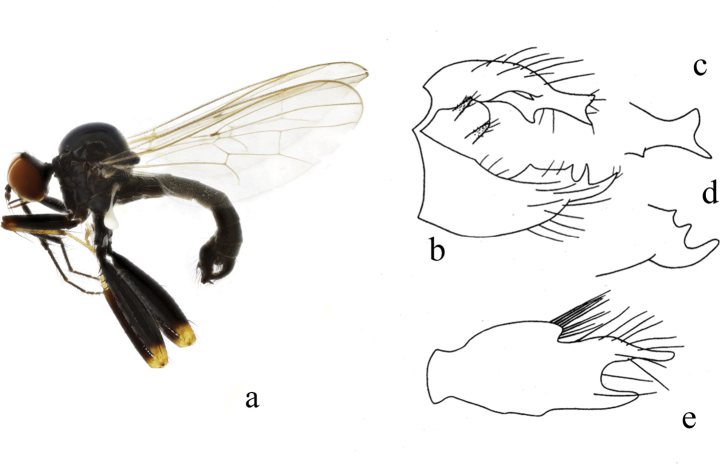
*Hybosparticularis***a** male habitus, lateral view **b** genitalia, dorsal view **c** right surstylus **d** left surstylus **e** hypandrium, ventral view (**b–e**: after Yang and Yang 2004)

#### Distribution.

China (Guangxi, Hainan); Thailand.

### ﻿ Hybos pingbianensis

Taxon classificationAnimaliaDipteraHybotidae

Yang & Yang, 2004

20CE3E70-3997-5E4D-8898-05B25E4A0F74

Fig. 18

Hybos pingbianensis Yang & Yang, 2004: 207

#### Type locality:

China: Yunnan, Pingbian, Daweishan.

#### Material examined.

China • 1♂, Guangxi, Guilin, Huaping, Anjiangping; 1413 m, 7 August 2023; Wenqiang Cao; CAU.

#### Diagnosis.

Legs yellow; hind coxae black; hind trochanter and femur black, hind tibia (except basal portion) blackish; tarsi dark brown, except fore and mid tarsomeres 1–2 and hind tarsomere 1 yellow. Right and left surstyli with three processes.

**Figure 18.**
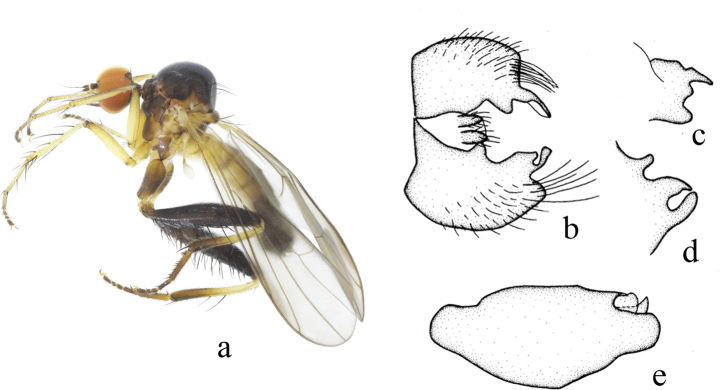
*Hybospingbianensis***a** male habitus, lateral view **b** genitalia, dorsal view **c** right surstylus **d** left surstylus **e** hypandrium, ventral view (**b–e**: after Yang and Yang 2004)

#### Distribution.

China (Guangxi, Yunnan).

### ﻿ Hybos serratus

Taxon classificationAnimaliaDipteraHybotidae

Yang & Yang, 1992

FC53598E-2CA8-5207-A9AE-590E211D9CC1

Fig. 19

Hybos serratus Yang & Yang, 1992: 1089; Yang and Yang 2004: 210.

#### Type locality.

China: Sichuan, Xichang.

#### Material examined.

China • 2♂, Guangxi, Guilin, Huaping, Anjiangping; 1413 m, 7 August 2023; Wenqiang Cao; CAU.

#### Diagnosis.

Legs yellow, except coxae yellow-brown, femora dark yellow, tarsomeres 2–5 dark yellow. Hypandrium large and wide with apical margin weakly incised medially

**Figure 19.**
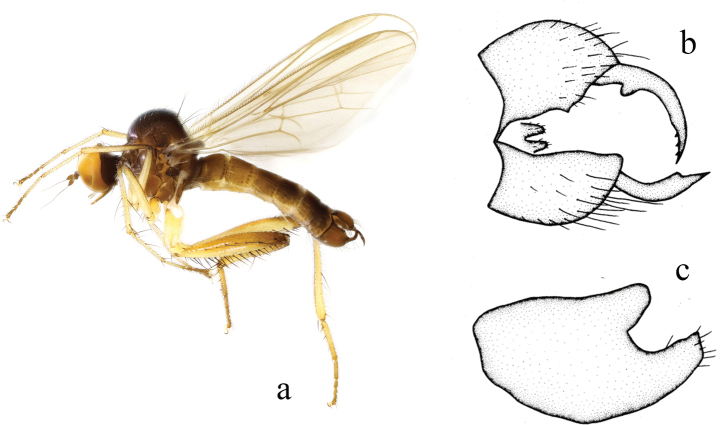
*Hybosserratus***a** male habitus, lateral view **b** genitalia, dorsal view **c** hypandrium, ventral view (**b, c**: after Yang and Yang 2004)

#### Distribution.

China (Fujian, Guangxi, Gzuihou, Henan, Sichuan, Yunnan, Zhejiang); Thailand

### ﻿ Hybos truncatus

Taxon classificationAnimaliaDipteraHybotidae

Yang & Yang, 1986

D50EF11D-0578-5332-B48D-75854D1737A9

Fig. 20

Hybos truncatus Yang & Yang, 1986: 80; Yang and Yang 2004: 220.

#### Type locality.

China: Guangxi, Longsheng.

#### Diagnosis.

Legs brownish. Mid tibia with one dorsal bristle at base, two long thin dorssal bristles at middle; apically with one long thin ventral bristle. Hypandrium large and wide, apical margin obliquely subtruncate with row of long bristles.

**Figure 20.**
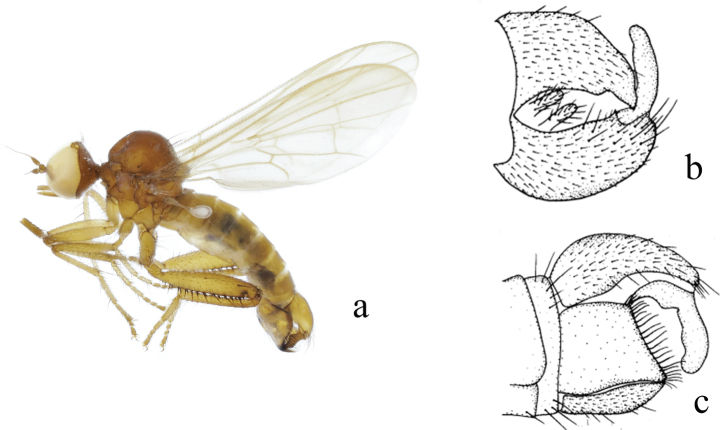
*Hybostruncatus***a** male habitus, lateral view **b** genitalia, dorsal view **c** hypandrium, ventral view (**b, c**: after Yang and Yang 2004)

#### Distribution.

China (Guangxi).

### ﻿ Hybos xiaohuangshanensis

Taxon classificationAnimaliaDipteraHybotidae

Yang, Gaimari & Grootaert, 2005

EC97882A-CCDF-5BF3-ABFA-62EE9FF57D27

Fig. 21

Hybos xiaohuangshanensis Yang, Gaimari & Grootaert, 2005: 5.

#### Type locality.

China: Guangdong, Nanling.

#### Material examined.

China • 3♂2♀, Guangxi, Guilin, Huaping, Anjiangping; 1340 m, 28 May 2023; Wei Zeng; CAU. China • 1♂, Guangxi, Guilin, Huaping, Hongtan; 849 m, 30 May 2023; Wei Zeng; CAU.

#### Diagnosis.

Arista bare. Legs black except hind knee (distal femur and proximal tibia) and mid and hind tarsi yellow-brown. Hypandrium obliquely incised apically, with long marginal bristles.

**Figure 21.**
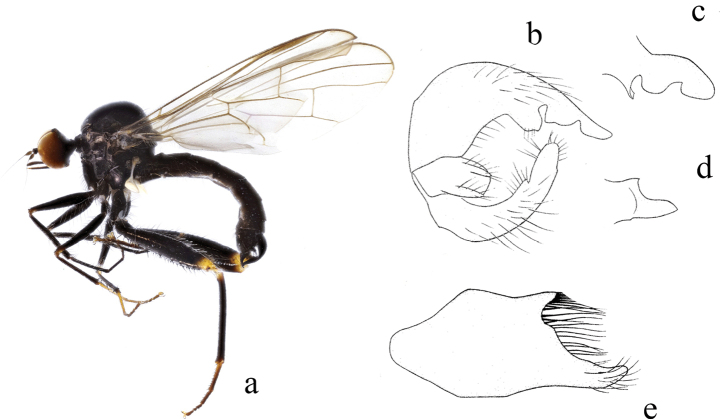
*Hybosxiaohuangshanensis***a** male habitus, lateral view **b** genitalia, dorsal view; **c** right surstylus **d** left surstylus **e** hypandrium, ventral view (**b–e**: after Yang, Gaimari and Grootaert 2005)

#### Distribution.

China (Fujian, Guangdong, Guangxi).

## ﻿Discussion

The main interspecific diagnostic characteristics in this genus include the short pubescent or bare arista, the color of the legs, the position of bristles on the legs, the relationship between R_4+5_ and M_1_ apically, and the shape of the hypandrium. Sexual dimorphism frequently occurs in *Hybos*, particularly in groups with yellow legs. The diverse female genitalia have also been identified as important specific characteristics (Plant 2013; Li and Yang 2023). Unfortunately, the females of the new species mentioned in the article have not been collected yet. They will be collected more extensively in the future for further study and supplementation.

Huaping National Nature Reserve is part of the Nanling Mountain range. Nanling Mountain Area is the largest mountain system and an important geographical boundary in southern China. It is also the largest oasis around 25 degrees north latitude and has a high diversity of flora and fauna. Two studies on local species richness in the family Argentidae (Hymenoptera) and butterflies (Lepidoptera) revealed that the insect fauna was predominantly composed of Oriental elements (You 2009; Zhou et al. 2016). This is consistent with the research findings of the article, where all nine new record species are from the Oriental region.

Huaping National Nature Reserve is a typical subtropical monsoon climate, with vegetation belonging to the category of evergreen broad-leaved forests. During the period we investigated from May to August, it was the rainy season, and the weather was mostly very humid. In the collected specimens, *Hybosparticularis* is widely distributed in Thailand but is often found in seasonal dry forest biotopes. This provides an interesting example for further exploration of the habitat of *Hybos*. This genus is species-rich and widely distributed in various ecoregions in China. Further research on *Hybos* biology, phenology, distribution patterns, and endemicity would be valuable and meaningful.

## Supplementary Material

XML Treatment for Hybotidae

XML Treatment for Hybos

XML Treatment for Hybos anae

XML Treatment for Hybos bawanglingensis

XML Treatment for Hybos chinensis

XML Treatment for Hybos denticulatus

XML Treatment for Hybos ensatus

XML Treatment for Hybos flaviscutellum

XML Treatment for Hybos forcipata

XML Treatment for Hybos fujianensis

XML Treatment for Hybos guizhouensis

XML Treatment for Hybos jianyangensis

XML Treatment for Hybos leucopogus

XML Treatment for Hybos longshengensis

XML Treatment for Hybos nasutus

XML Treatment for Hybos obtusatus

XML Treatment for Hybos orientalis

XML Treatment for Hybos paraterminalis

XML Treatment for Hybos particularis

XML Treatment for Hybos pingbianensis

XML Treatment for Hybos serratus

XML Treatment for Hybos truncatus

XML Treatment for Hybos xiaohuangshanensis

## ﻿Additional information

### Author ORCIDs

Meilin Li https://orcid.org/0000-0003-2473-110X

Jingyu Wang https://orcid.org/0009-0009-0396-8547

Ding Yang https://orcid.org/0000-0002-7685-3478

### Data availability

All of the data that support the findings of this study are available in the main text.
